# Ductility and Strength Reduction Factors for Degrading Structures Considering Cumulative Damage

**DOI:** 10.1155/2014/575816

**Published:** 2014-04-22

**Authors:** Edén Bojórquez, Sonia E. Ruiz, Alfredo Reyes-Salazar, Juan Bojórquez

**Affiliations:** ^1^Facultad de Ingeniería, Universidad Autónoma de Sinaloa, Calzada de las Américas y Boulevard Universitarios S/N, Ciudad Universitaria, 80040 Culiacán Rosales, SIN, Mexico; ^2^Coordinación de Mecánica Aplicada, Instituto de Ingeniería, Universidad Nacional Autónoma de México, Ciudad Universitaria, Coyoacán, 04510 México, DF, Mexico

## Abstract

The effect of cumulative damage on the strength requirements of degrading structures is assessed through the evaluation of the target ductility and corresponding strength reduction factors of simple degrading structures. While the reduction on ductility is established through the use of Park and Ang index, the suggestions given by Bojórquez and Rivera are used to model the degradation of the structural properties of the simple systems. Target ductilities and their corresponding reduced strength reduction factors are established for five sets of ground motions; most of them are recorded in California. The results given in this paper provide insight into all relevant parameters that should be considered during seismic design of earthquake-resistant structures. Finally, some recommendations to evaluate the effect of cumulative damage on seismic design are suggested.

## 1. Introduction 

A structure subjected to several cycles of plastic behavior can exhibit excessive degradation of its structural properties and, as a consequence, failure at deformation levels that are significantly smaller than the one developed during monotonically increasing deformation. Under these circumstances, it is necessary to incorporate into seismic design information that allows for a numerical characterization of the severity of cumulative plastic deformation demands. Within the format of most current seismic design codes, a fundamental tool to establish the strength requirements of an earthquake-resistant structure is the use of response spectra, obtained from the response of nonlinear single degree of freedom (SDOF) systems. One practical way to incorporate cumulative plastic deformation demands into seismic design is the formulation of strength reduction factors that take into account the effect of cumulative damage through the definition of a reduced or target ductility [1]. Currently, the incorporation of cumulative damage on strength reduction factors through the concept of target ductility has been addressed for SDOF with elastoplastic behavior [2]. However, structures used for engineering purposes usually exhibit strength and stiffness degradation under the effect of cyclic loading. Within this context, the use of elastoplastic behavior can result in unrealistic modeling and inadequate estimation of seismic demands. Particularly, in some cases, such as degrading systems subjected to severe cumulative demands or long-duration motions, significant underestimation of earthquake effects can occur when using nondegrading models to characterize the seismic demands [3, 4]. Because many studies have shown the influence of ground motion duration on structural response and performance [1, 5–9], this paper is aimed at estimating the target ductility and corresponding strength reduction factor for degrading structures. While the estimation considers the effect of cumulative plastic demands through the Park and Ang damage index, five sets of ground motions, most of them recorded in California, were used. The implication of using other damage indices is also assessed. The results shown in this paper give an insight into all relevant parameters that should be incorporated into the seismic design of structures subjected to earthquake ground motions and provide a basis under which qualitative and quantitative recommendations can be formulated to take into account the effect of cumulative damage in the design of degrading structures.

## 2. Degradation Function and Structural Models 

**Figure 1:**
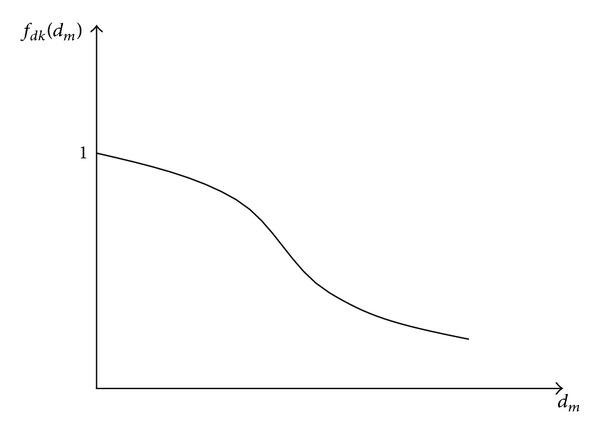
Stiffness degradation function for a system based on maximum displacement demand.

Several hysteretic models have been proposed to represent the behavior of structures that exhibit strength and stiffness degradation [10, 11]. This paper does not focus on describing such models, but on illustrating the effect of having some specific conditions in the constitutive laws that model the cyclic behavior of degrading systems. A function *f*_*d*_ can be formulated to describe the degradation of a specific structural property of a system in terms of a specific performance parameter (e.g., maximum displacement) and can be used to estimate the strength and/or stiffness of the system at any instant of time in terms of the initial values of these properties.  Figure 1 illustrates a function *f*_*dk*_(*d*_*m*_) for the stiffness *k* of a system in terms of the maximum displacement *d*_*m*_. While an increase in the maximum displacement results in a reduction of stiffness, *f*_*dk*_(*d*_*m*_) = 1 for *d*_*m*_ = 0. The stiffness at a specific stage is given by *k* = *f*_*dk*_(*d*_*m*_)*k*_*o*_, where *k*_*o*_ represents the initial stiffness of the system.

**Figure 2:**
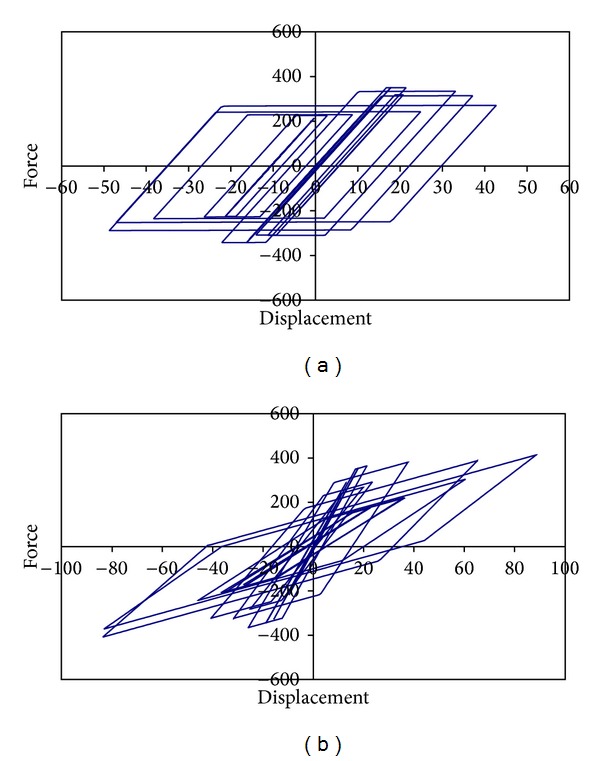
Hysteretic model with degradation in (a) strength and (b) strength and stiffness.

The use of a degradation function based on maximum displacement results in some circumstances in an underestimation of the loss of strength and stiffness. Particularly, the degradation of strength and stiffness typically observed during the experimental testing of structural elements that undergo severe cumulative plastic demands show the limitations of displacement-based degrading models. After analyzing the results derived from experimental testing of steel and concrete elements, Bojórquez and Rivera [12] concluded that the use of degradation functions based on peak parameter response can be inadequate. Under these circumstances, it is important to use parameters that can adequately characterize the actual level of degradation exhibited by structures subjected to cyclic loads. This paper considers SDOF systems by an interval periods from 0.1 to 5 sec and exhibit elastoplastic (EP) behavior with different strain-hardening and 5% of critical damping. In some cases, the EP models are degraded with the aid of a function that modifies the initial strength (*F*_*yo*_) and displacement (*d*_*yo*_) at yield as a function of the normalized dissipated hysteretic energy [12]. As shown in Figure 2, a diversity of hysteretic models can be obtained under these considerations.

The general form of the degradation function used in this paper to assess the effect of cumulative demands is given by Bojórquez and Rivera:

(1) $$\begin{array}{c} f_{d}\left(I_{D}\right)=\;{\left(\frac{a}{a+I_{D}}\right)}^{\gamma }, \end{array}$$

where *I*_*D*_ represents a damage parameter and *a* and *γ* are constants. For a specific value of *a*, the rate of degradation of the system will depend on the value assigned to *γ*. Due to the high correlation that exists between the normalized dissipated hysteretic energy and the level of structural damage [3], it is convenient to express (1) in terms of normalized dissipated hysteretic energy:

(2) $$\begin{array}{c} f_{d}=\;{\left(\frac{E_{\text{NC}}}{E_{\text{NC}}+E_{\text{ND}}}\right)}^{\gamma }, \end{array}$$

where *E*_NC_ is the normalized hysteretic energy capacity of the system and *E*_ND_ its corresponding demand. Equation (2) is used in this paper to characterize the level of strength and stiffness degradation.

To represent the global hysteretic behavior of different types of structures, this paper uses three sets of values for *E*_NC_ and *γ* to represent three levels of degradation [12]: (i) no degradation; (ii) stiffness and strength degradations of about 10% and 20%, respectively, which are typical of steel elements; and (iii) stiffness and strength degradations of about 30% and 20%, which are typical of reinforced concrete elements.  Table 1 summarizes the hysteretic behavior models under consideration.

## 3. Damage Indices

The selection of a particular damage index to formulate analytical studies should be done carefully. On one hand, all damage indices have limitations, and, because of this, the results derived from their use must be carefully interpreted. On the other hand, the use of damage indices formulated on significantly different terms results in similar strength demands when cumulative plastic deformation demands are explicitly taken into consideration [3]. At the end, there is an adequate level of certainty around the trends and design implications derived from the use of a specific damage index. In this paper, the Park and Ang damage index was used because it is well known and is adequately supported by extensive experimental and analytical calibration [13, 14].

According to Park and Ang [13], the level of structural damage in concrete elements and structures subjected to cyclic loads can be estimated through the linear combination of their maximum and cumulative demands:

(3) $$\begin{array}{c} I_{\text{DPA}}=\frac{\mu_{m}}{\mu }+\beta \frac{E_{H}}{F_{y}d_{y}\mu }, \end{array}$$

where *μ*_*m*_ is the maximum ductility developed by the system when subjected to an earthquake motion; *μ* is the relation between the ultimate displacement under monotonic deformation and the displacement at first yield (ultimate ductility); *β* is a parameter to characterize the stability of the hysteretic cycle; and finally, *F*_*y*_ and *d*_*y*_ are the force and displacement at first yield, respectively. A *β* value of 0.15, considered to represent the behavior of structures with adequate seismic detailing [15], was used for the EP and B5D10 models; *β* = 0.30, considered to represent the case of systems with important level of degradation [14], was used in the case of the B5D30 model. Theoretically, *I*_DPA_ equal to zero represents no damage and a value of one is associated to the failure of the system.

The target or maximum ductility in the structure associated with incipient failure (*I*_DPA_ = 1) is given by Fajfar [1]:

(4) $$\begin{array}{c} \mu_{m}=\frac{\sqrt{1+4\beta \gamma^{2}\mu }-1}{2\beta \gamma^{2}}, \end{array}$$

where

(5) $$\begin{array}{c} \gamma =\frac{\sqrt{E_{H}/m}}{\omega d_{m}} \end{array}$$

and *m* is the mass of the system, *d*_*m*_ is the maximum displacement, and *ω* is the circular frequency. Equation (4) indicates that the maximum ductility demand is controlled by the values of parameters *β* and *γ* and of the ultimate ductility *μ*.

It should be emphasized that several studies, such as those carried out by Cosenza and Manfredi [16] and Terán-Gilmore and Jirsa [3], have shown that, under the consideration of the effects of the cumulative plastic deformation demands, the strength requirements derived from the use of the Park and Ang damage index are very similar to those derived from other damage indices, such as the one based on Miner's Hypothesis and the Teran and Jirsa damage index.

## 4. Earthquake Ground Motion Records

**Figure 3:**
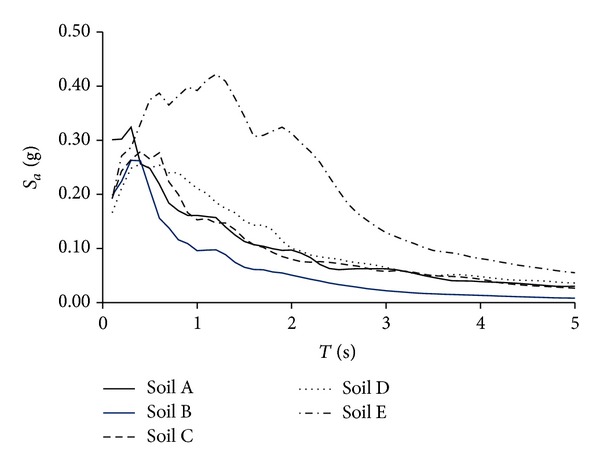
Average spectra for the selected ground motion records sets.

Five sets of twenty earthquake ground motions obtained from the Next Generation Attenuation (NGA) database were used. All records were selected based on Geomatrix site classes (GMX's C3). Particularly, the five sets correspond to soil types A, B, C, D, and E, which represent rock, stiff, deep narrow, deep broad, and soft soil conditions, respectively. Most of the selected records correspond to California and have earthquake magnitudes larger than 6.0.  Figure 3 illustrates the average pseudoacceleration response spectra (*S*_*a*_) for each set of ground motions.

## 5. Maximum Ductility Results

**Figure 4:**
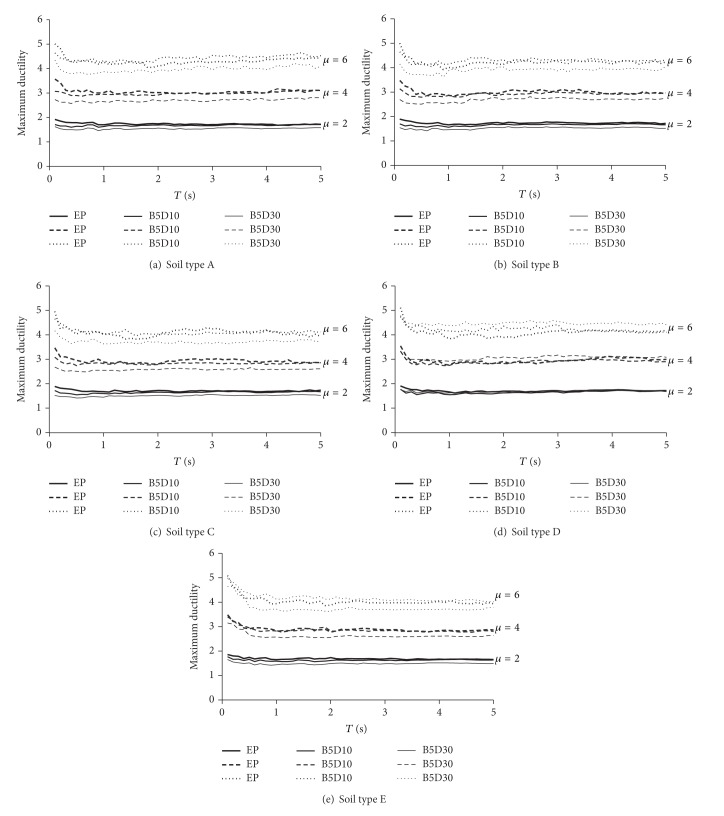
Average target ductility for different hysteretic models and ultimate ductilities.

Figure 4 summarizes the target ductilities for all sets of motions, hysteretic models, and ultimate ductilities under consideration. The figure plots the value of the average target ductility as a function of the structural period, level of degradation and ultimate ductility of the SDOF systems, and the type of soil. Note that the target ductility is practically independent of the structural period, type of soil, and level of degradation. Also, the target ductilities corresponding to models EP and B5D10 are very similar for all types of soil and values of ultimate ductility; this fact strongly suggests that the global behavior of all steel structures can be modeled in a reasonable manner through the use of an elastoplastic model with no degradation. Particularly, the use of a nondegrading elastoplastic model results in an adequate evaluation of the effects of cumulative damage in the value of the target ductility of degrading steel systems. In the case of the B5D30 model, which represents the behavior of reinforced concrete structures, the target ductilities exhibit slightly smaller values than those corresponding to the EP model. In general and for different values of ultimate ductilities and types of soil, the average target ductility for the B5D30 model tends to be 10% smaller than that obtained for the EP model.

**Figure 5:**
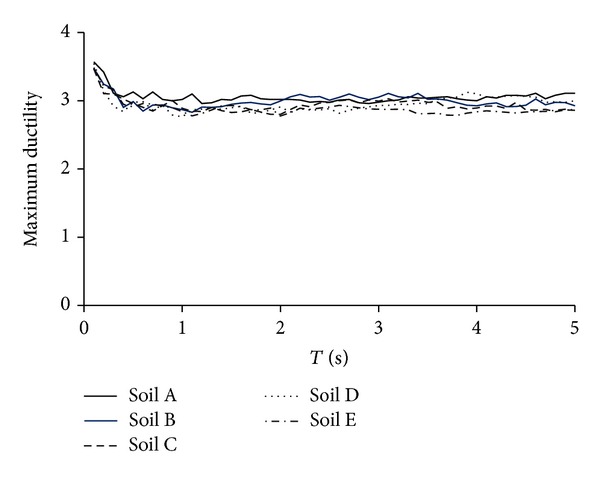
Influence of type of soil in the target ductility for *μ* = 4 (EP model).

**Figure 6:**
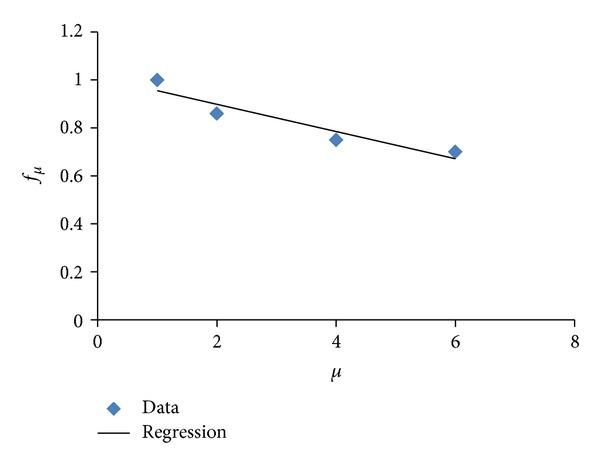
Evaluation of *f*_*μ*_ with respect to *μ*.

Figure 5 shows no significant influence of the type of soil on the value of the target ductility. Particularly, the results summarized in the figure suggest that the target ductility can be estimated from a simple relation, such as *μ*_*m*_^*EP*⁡^ = *f*_*μ*_ *μ*, where *f*_*μ*_ is a parameter that reduces the maximum ductility demand with respect to the ultimate ductility capacity to take into account the cumulative plastic deformation demands and *μ*_*m*_^*EP*⁡^ is the target ductility associated with incipient failure of a system exhibiting EP behavior. A regression analysis (see Figure 6) show values from 0.7 to 0.95 for *f*_*μ*_; depending on the ultimate ductility, it suggests that the ultimate ductility capacity should be reduced up to 30% to estimate the maximum ductility that can be undergone by earthquake-resistant structures during severe shaking. Note that the target ductility for highly degrading systems (*μ*_*m*_^DEG^) can be estimated as 0.9*μ*_*m*_^*EP*⁡^.

## 6. Strength Reduction Factors Considering Cumulative Damage

Although strength reduction factors (*R*_*μ*_) are estimated in this section for the EP model, it should be emphasized that the results previously discussed indicate no significant influence of the hysteretic model on the strength requirements of the SDOF systems under consideration in this paper. Within this context, it should be considered that the values of *R*_*μ*_ shown herein are applicable to models EP and B5D10 and that strength reductions factors for model B5D30 should be increased in 10% with respect to the previous values.

Only results for soil type B are shown. Within this context, it should be recalled that no significant influence of the type of soil was observed in the strength requirements of the SDOF systems under consideration.

**Figure 7:**
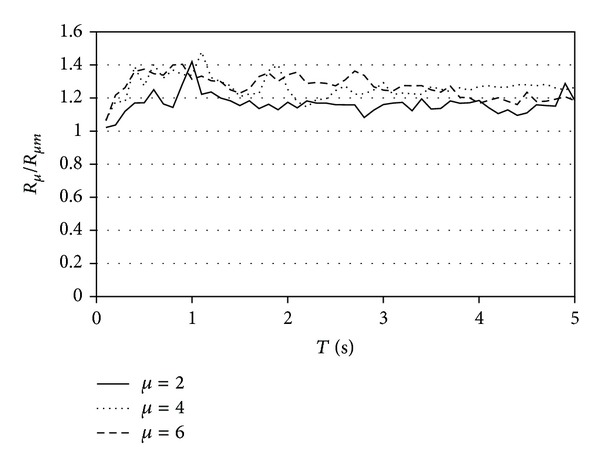
Average ratio between *R*_*μ*_ and *R*_*μm*_ for soil type B.

Figure 7 illustrates the ratio between *R*_*μ*_ and *R*_*μm*_ for systems exhibiting EP behavior. While both *R*_*μ*_ and *R*_*μm*_ represent strength reduction factors, the former factor corresponds to the case in which no explicit consideration is made for the effect of cumulative plastic demands and the latter factor to the case in which this effect is taken into consideration. Within this context, *R*_*μ*_ is the ratio of the minimum strength required to maintain the structure elastic during the ground motion and that required to limit its ductility demand within the threshold defined by the value of the ultimate ductility capacity *μ*_*i*_:

(6) $$\begin{array}{c} R_{\mu }=\frac{Sa\left(\mu =1\right)}{Sa\left(\mu =\mu_{i}\right)}. \end{array}$$

Equation (6) is the basis in which the majority of current seismic design codes establish design strength requirements for earthquake-resistant structures that develop different level of nonlinear behavior. The concept of *R*_*μm*_ is similar, except that the threshold for the maximum ductility demand in the system does not correspond to its ultimate ductility capacity, but to the value of the target ductility *μ*_*mi*_:

(7) $$\begin{array}{c} R_{\mu m}=\frac{Sa\left(\mu =1\right)}{Sa\left(\mu ={\mu_{m}}_{i}\right)}. \end{array}$$

Figure 7 shows that the ratio *R*_*μ*_/*R*_*μm*_ practically exhibits a constant value with respect to those of the period and ultimate ductility capacity of the SDOF systems. The results summarized in the plot suggest that, to take into account explicitly the effects of cumulative damage, strength reduction factors should be reduced by around 30% with respect to those currently used during seismic design.

## 7. Conclusions 

The influence of cumulative plastic deformation demands on the values of the target ductility and their corresponding strength reduction factors was studied. The results shown in this paper strongly suggest that, in order to account explicitly for the effect of cumulative plastic demands, the maximum or target ductility that an earthquake-resistant structure can undergo during a ground motion should be limited within a threshold defined by 0.7 of its corresponding ultimate ductility capacity. This conclusion is valid for any system exhibiting no or moderate degradation of its hysteretic behaviour during cyclic loading and located in a wide range of soil conditions. In the case of a system exhibiting significant degradation of its structural properties, its target ductility should be further reduced by 10% with respect to the value corresponding to a nondegrading system.

Finally, it is important to mention that the results presented herein may not be valid for soil conditions that differ from those under consideration in the paper. Particularly, long-duration narrow-banded motions, such as those generated in the Lake Zone of Mexico City, require the formulation of specific studies and recommendations.

## Figures and Tables

**Table 1:** Hysteretic behavior models under consideration.

Hysteretic model	Postyielding stiffness (%)	*γ* (strength degradation)	*γ* (stiffness degradation)	*E*_NC_	Description
EP	0	0	0	9	Nondegrading EP model
B5D10	5	0.321	0.152	7.5	Strength degradation and low stiffness degradation typical of steel structures
B5D30	5	0.321	0.515	5.6	Strength degradation and moderate to large stiffness degradation typical of reinforced concrete structures
